# Novel Microsatellite Markers Acquired from *Rubus coreanus* Miq. and Cross-Amplification in Other *Rubus* Species

**DOI:** 10.3390/molecules20046432

**Published:** 2015-04-10

**Authors:** Gi-An Lee, Jae Young Song, Heh-Ran Choi, Jong-Wook Chung, Young-Ah Jeon, Jung-Ro Lee, Kyung-Ho Ma, Myung-Chul Lee

**Affiliations:** 1 National Agrobiodiversity Center, National Academy of Agricultural Science, Rural Development Administration, Jeonju 560-500, Korea; E-Mails: gkntl1@korea.kr (G.-A.L.); jysong77@korea.kr (J.Y.S.); jwchung73@korea.kr (J.-W.C.); yjeon@korea.kr (Y.-A.J.); jrmail@korea.kr (J.-R.L.); khma@korea.kr (K.-H.M.); 2 Black Raspberry R&D Team, Gochanggun Agricultural Extension Center, Gochang, Jeollabuk-do 585-943, Korea; E-Mail: hehranchoi@korea.kr

**Keywords:** microsatellite, genetic diversity, transferability, *Rubus* genus

## Abstract

The *Rubus* genus consists of more than 600 species that are distributed globally. Only a few *Rubus* species, including raspberries and blueberries, have been domesticated. Genetic diversity within and between *Rubus* species is an important resource for breeding programs. We developed genomic microsatellite markers using an SSR-enriched *R. coreanus* library to study the diversity of the *Rubus* species. Microsatellite motifs were discovered in 546 of 646 unique clones, and a dinucleotide repeat was the most frequent (75.3%) type of repeat. From 97 microsatellite loci with reproducible amplicons, we acquired 29 polymorphic microsatellite markers in the *Rubus coreanus* collection. The transferability values ranged from 59.8% to 84% across six *Rubus* species, and *Rubus* *parvifolius* had the highest transferability value (84%). The average number of alleles and the polymorphism information content were 5.7 and 0.541, respectively, in the *R. coreanus* collection. The diversity index of *R. coreanus* was similar to the values reported for other *Rubus* species. A phylogenetic dendrogram based on SSR profiles revealed that seven *Rubus* species could be allocated to three groups, and that *R. coreanus* was genetically close to *Rubus crataegifolius* (mountain berry). These new microsatellite markers might prove useful in studies of the genetic diversity, population structure, and evolutionary relationships among *Rubus* species.

## 1. Introduction

The *Rubus* genus in the Rosaceae family consists of more than 600 species grouped in 12 subgenera. A few of these species, including raspberries, blackberries, dewberries, arctic fruits, and flowering raspberries, have been domesticated and are the focus of breeding programs [1]. Both cultivated and wild *Rubus* species have the potential to interact with other species belonging to different *Rubus* subgenera. Cross-fertilization within the *Rubus* genus implies that wild populations could be useful resources to improve domesticated species [2,3,4]. For this reason, *Rubus coreanus*, which is distributed throughout Southeast Asia, could be a valuable resource for breeding programs and the biotech industry [5,6].

During the last few decades, molecular markers have been used to estimate the genetic diversity of *Rubus* populations. Studies focusing on the genetic diversity of *Rubus* species have been conducted using various types of molecular markers, including restriction fragment length polymorphisms, random amplified polymorphic DNA, simple sequence repeats (SSRs), and amplified fragment length polymorphisms (AFLP), in *Rubus caucasicus* L. [7], *Rubus glaucus* [8], *Rubus idaeus* [9,10], *Rubus occidentalis* [11,12], and other *Rubus* species [13,14,15]. *Rubus idaeus* and *Rubus caesius* hybrids have been surveyed using internal transcribed spacer markers [16]. Cross-amplification within these *Rubus* species involved AFLP and SSR markers [15].

More reliable molecular markers are needed to enhance genetic analyses of *Rubus* species. The objectives of this study were to develop novel genomic SSR markers using a microsatellite-enriched library of *R. coreanus* and to test their transferability across six other *Rubus* species. New microsatellite markers with polymorphisms and transferability across species might be valuable tools to evaluate genetic variability and identify genes controlling agronomic traits in the *Rubus* genus.

## 2. Results and Discussion

In this study, 684 positive colonies were randomly selected from an SSR-enriched library and sequenced. A total of 358 kb sequences were acquired in 684 clones, and 646 singleton sequences were used to search for SSR motifs. A total of 38 (5.6%) of the 684 clones were duplicates. Microsatellite motifs were discovered in 546 clones. The enrichment efficiency of microsatellites (84.5%) was higher than for groundnut at 68% [17], Japanese apricot at 57.0% [18], lychee at 52.0% [19], and *Actinidia arguta* at 74.2% [20].

Most of the microsatellite motifs (75.3%) were dinucleotide repeats, followed by trinucleotide repeats (20.7%) and repeat motifs that were tetranucleotide or greater (4.0%). Among the SSR motifs, AG/CT and CTT/AAG represented the majority of di- and trinucleotide motifs at 57.1% and 6.0%, respectively. This result corresponds to the general SSR motif distribution in dinucleotide motifs [21,22]. The most common repeat in plants is the AT repeat; however, it was rare (1.1%) in this study. The AT motif is not suitable for hybridization, due to its autocomplementarity [23].

Among the 546 clones containing microsatellite motifs, 263 primer pairs were designed based on flanking SSR regions. The SSR motifs biased over the acquired sequences might affect primer variation. The divergent distribution of DNA fragments in the SSR-enriched library could introduce variation among crops [24]. In total, 97 (46.2%) of the microsatellite markers covered by the 263 primer pairs were successfully amplified and had reproducible amplicons (Supplementary Table S1); the remaining markers had no amplicons or multi-bands between two *R. coreanus* accessions, and were thus excluded from further analysis.

We applied the 97 microsatellite markers to analyze their transferability to other *Rubus* species (Table 1). The results for six other *Rubus* species indicated that, except for eleven loci with no amplified products, 86 (88.7%) of the microsatellite markers produced at least one amplicon in other *Rubus* species. A total of 26 (26.8%) of the microsatellite markers had PCR products in all tested samples (Table 2 and Supplementary Table 1). The transferability values ranged from 59.8% to 84% across six *Rubus* species. *Rubus parvifolius* had the highest transferability value (84%), followed by *Rubus ursinus* (79.4%) and *R. idaeus* (78.4%); in contrast, *Rubus crataegifolius* had only 59.8% transferability.

**Table 1:** List of accessions belonging to the *Rubus* genus.

*Rubus* Species (Sample Size)	Accession no.
*R. coreanus* (32)	GCB0021 *, GCB0023, GCB0024, GCB0027, GCB0029, GCB0030, GCB0032, GCB0033 *, GCB0034, GCB0035, GCB0036, GCB0038, GCB0040, GCB0041, GCB0042, GCB0043, GCB0045, GCB0046, GCB0047, GCB0049, GCB0051, GCB0052, GCB0054, GCB0055, GCB0057, GCB0059, GCB0060, GCB0061, GCB0062, GCB0063, GCB0118, GCB0119
*R. crataegifolius* var. subcuneatus (3)	GCB0001, GCB0002, GCB0120
*R. parvifolius* (2)	GCB0004, GCB0005
*R. crataegifolius* (4)	GCB0006, GCB0009, GCB0014, GCB0015
*R. ursinus* (1)	GCB0066
*R. fruticosus* (3)	GCB0067, GCB0068, GCB0069
*R. idaeus* (3)	GCB0071, GCB0072, GCB0073

* Accessions used to check for amplicon production using the designed primer pairs.

**Table 2:** Transferability of 97 new microsatellite markers within the *Rubus* genus.

	*Rubus Species*						
	R. co *	R. p *	R. i *	R. u *	R. f *	R. cs *	R. c *
Mean transferability (%)	100	84 (83.5–84.5)	78.4 (75.3–80.4)	79.4	71.8 (66.0–76.3)	67.7 (63.9–71.1)	59.8 (56.7–61.9)

* Abbreviation: R.co, *R. coreanus*; R.cs, *R. crataegifolius* var. subcuneatus; R.p, *R. parvifolius*; R.c, *R. crataegifolius*; R.u, *R. ursinus*; R.f, *R. fruticosus*; R.i, *R. idaeus*.

Among the transferable microsatellite markers, we identified 29 polymorphic markers in selected *R. coreanus* accessions, while the remaining markers were monomorphic. These 29 markers were used to create DNA profiles of 48 *Rubus* accessions (*R. coreanus*, 32 accessions; other *Rubus* species, 16 accessions). Polymorphism scores for 29 microsatellite markers were calculated for the *R. coreanus* collection and for other *Rubus species* (Table 3). The number of alleles (N_A_) varied among loci, and ranged from two to 13 (mean = 5.7 alleles) alleles in *R. coreanus* and from four to 15 (mean = 9.6 alleles) alleles in other *Rubus* species. Within the *R. coreanus* collection, the mean expected heterozygosity and observed heterozygosity were 0.582 and 0.558, respectively, and the mean polymorphism information content (PIC) was 0.541 (range, 0.147–0.863). GB-RC-245 had the highest PIC value of 0.863 (N_A_ = 13), followed by 0.826 for GB-RC-091 and GB-RC-247 (N_A_ = 8 and 10, respectively). Castillo *et al.* [25] reported 12 microsatellite markers from red raspberry (*R. idaeus*) and blackberry (*Rubus* L. hybrids), with an average PIC value of 0.55 in the red raspberry collection. Michael *et al.* [26] reported an average PIC value of 0.49 (mean N_A_ = 8.6) using 21 polymorphic *Rubus* SSR primers in cultivated and wild black raspberry (*R. occidentalis* L.) collections. Our results revealed a similar diversity index in the *R. coreanus* collection compared to previously reported microsatellite markers in other *Rubus* species. This implies that the microsatellite markers developed in this study might be useful for the genetic assessment of *Rubus* species with high transferability.

**Figure 1:**
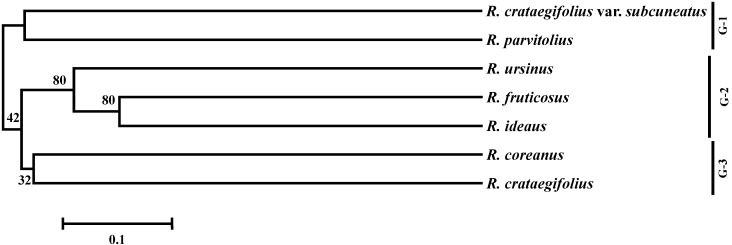
Phylogentic dendrogram based on SSR profiles in *Rubus* species.

Microsatellite analyses of *R. coreanus* and the genetic relatedness among seven *Rubus* species based on SSR profiles were not previously reported, whereas several studies have focused on cultivated *Rubus* species such as red raspberry [10,25]. In this study, we constructed phylogenetic trees based on the DNA profiles of 48 *Rubus* accessions using 29 microsatellite loci. The *Rubus* species were divided into three groups (Figure 1). *Rubus coreanus* was genetically close to mountain berry (*R. crataegifolius*) in the third group (G-3). The alleles of *R. crataegifolius* for the 29 microsatellite loci were similar to those of *R. coreanus*, although *R. crataegifolius* had low transferability (59.8%). The first group (G-1) consisted of other mountain berries (*R. crataegifolius* var. *subcuneatus* and *R. parvifolius*), while the second group (G-2) included blackberry (*Rubus fruticosus* and *R. ursinus*) and red raspberry. These microsatellite markers will be useful for studies of the genetic diversity, population structure, and evolutionary relationships among *Rubus* species. 

**Table 3:** Summary of the 29 polymorphic microsatellite markers in *Rubus coreanus*.

Primer Name	Genebank No	Primer Sequence (5'-3')	R-Motif	Size Range	*R. coreanus*				Other *Rubus* sp.		Tr *
					N_A_	H_O_	H_E_	PIC	N_A_	PIC	
GB-RC-020	JX976551	F-AAGCAATAATGGGTGGATCA R-AATGGGAAGGCTGCAACT	(GAA)9	303–318	4	0.607	0.696	0.647	8	0.809	50
GB-RC-049	JX976552	F-ACAAGGTTGGTGAATGCG R-ATTGCACTCTTCCGCTCA	(GA)4	196–210	2	0.333	0.278	0.239	5	0.745	31.3
GB-RC-062	JX976553	F-ACGACCCTTTGAATCGCT R-GCGAGGCAAGTATTGGTG	(ATG)5	157–175	3	0.25	0.279	0.255	4	0.639	37.5
GB-RC-067	JX976554	F-AGAAGGTGTGCGAGACCC R-AACCGTGTCACCGTGAAG	(AG)19	287–297	6	0.233	0.584	0.557	-	-	-
GB-RC-074	JX976555	F-AGAGTGGCCCTAGCCTTG R-ACCCGATGAAGCTGGTTT	(AG)15	210–226	6	0.613	0.482	0.446	10	0.815	93.8
GB-RC-077	JX976557	F-AGCACCCTCTAAACCCGA R-TGCTCATATATAATCGATGTGCTT	(AAC)9	194–208	6	0.742	0.552	0.508	7	0.565	93.8
GB-RC-078	JX976558	F-AGCAGCATCATCAGTTCCA R-TGCTTGGTGACCTCTGCT	(GCA)6	192–224	5	0.774	0.699	0.643	14	0.904	81.3
GB-RC-091	JX976559	F-ATCCCGAAAACCACCATT R-CCTCTCTCTCCCCGTGAA	(AG)15	235–259	8	0.281	0.845	0.826	11	0.844	81.3
GB-RC-098	JX976560	F-ATGCCTCGATTGCAGAGA R-GAACTCACAGCAGGTCGC	(GCA)4	201–216	3	0.167	0.155	0.147	4	0.683	100
GB-RC-100	JX976561	F-ATGTGCAGCAGCAGTGAA R-CTGGGTCCATCCACATTG	(AG)6	210–286	8	0.548	0.771	0.741	11	0.852	100
GB-RC-105	JX976562	F-ATTAAACCTCACCGGCGT R-CAAGGCTTGTCAATTCGG	(GT)8	167–183	4	0.969	0.626	0.566	9	0.798	93.8
GB-RC-109	JX976563	F-CAAGAGTTGCATCGGCTC R-TGTTGAAACTTTGCCATGC	(TC)12	169–195	6	0.387	0.614	0.538	15	0.893	87.5
GB-RC-111	JX976564	F-CAAGGCTTGTCAATTCGG R-ATTAAACCTCACCGGCGT	(CA)11	170–184	4	0.968	0.623	0.56	8	0.753	93.8
GB-RC-138	JX976565	F-CCACAAAACCAAGACCCA R-AAGATAGATGAGGCCAGCG	(ACACTC)4	213–220	4	0.469	0.471	0.387	11	0.861	93.8
GB-RC-141	JX976566	F-CCACAGAATGGGATTCATA R-CTCGACTTGTCGCAGAGG	(GA)6	163–189	4	0.281	0.249	0.231	12	0.872	87.5
GB-RC-143	JX976567	F-CCACGGAGGACGTAATGA R-CAGTCCAACTTGCTTCCG	(AG)12	207–225	6	0.719	0.625	0.564	10	0.81	62.5
GB-RC-145	JX976568	F-CCATATGACACAGCCCAAA R-CCATGCGACTTTACTGCC	(AC)9, (CAA)5	276–280	3	0.188	0.246	0.222	6	0.672	100
GB-RC-166	JX976570	F-CCTACGGCTTTGGTATGTT R-CCCCCTTTCTCCTTCCTT	(TTGAAG)6	181–207	5	0.625	0.751	0.71	12	0.871	93.8
GB-RC-167	JX976571	F-CCTCATTTGCAAAGGTTCT R-GACCGAACCATGATGGAA	(TC)17	187–247	12	0.759	0.819	0.796	14	0.884	81.3
GB-RC-178	JX976572	F-CGCGCTAAACCACTTCAC R-GTTGGAACAGCAGTGGGA	(CA)15	167–191	6	1	0.67	0.618	11	0.829	81.3
GB-RC-186	JX976573	F-CGTCCAATGTCTATCCGC R-CAGGCAACTGCGATCTTC	(TTG)5	269–295	6	0.621	0.69	0.635	11	0.87	81.3
GB-RC-191	JX976574	F-CTCAATGGGAGCACCAAA R-CCCTGCCCAATAAGCATT	(GA)6	275–287	7	0.862	0.797	0.772	12	0.803	93.8
GB-RC-193	JX976575	F-CTCCCTGCAAAGAAAGCC R-CTGGAATTCGCCCTTCTC	(GA)18	275–287	7	0.857	0.782	0.753	9	0.804	81.3
GB-RC-207	JX976577	F-CTTAGCCAGAACGGGGAG R-CTAACCGGCTGGCCTACT	(GA)10	219–231	6	0.643	0.577	0.512	-	-	-
GB-RC-211	JX976578	F-CTTGGTGTGGATGCGATT R-TTCCAGATTCGACCGTTG	(GGA)7	196–210	6	0.5	0.556	0.527	8	0.838	100
GB-RC-220	JX976579	F-GAATCAGGGTGAAGGGGA R-CCCCCTCTCTTCTTTTTGG	(GA)14	272–276	2	0.379	0.307	0.26	9	0.797	62.5
GB-RC-245	JX976582	F-GCCTCAAGCTCACACAGG R-GGTGCGTCCACAAACTGT	(AG)14	262–350	13	0.645	0.876	0.863	13	0.897	93.8
GB-RC-247	JX976583	F-GCGCTATGGTCAGGTTGA R-AAAGAAACGGTGGCCATT	(TTC)12	278–338	10	0.6	0.844	0.826	10	0.874	62.5
GB-RC-259	JX976584	F-GGAAGGAATGCAATAGCCA R-TCCCCCTGCTTCTGAGAT	(TG)8	315–317	2	0.161	0.425	0.335	5	0.704	43.8
Mean					5.7	0.558	0.582	0.541	9.6	0.803	80.1

* Abbreviations: N_A_, number of alleles; H_O_, observed heterozygosity; H_E_, expected heterozygosity; PIC, polymorphism information content.

## 3. Experimental Section

### 3.1. Plant Materials and Genomic DNA Extraction

DNA was extracted from 48 *Rubus* accessions (32 *R. coreanus* accessions and 16 accessions of other *Rubus* species) that were conserved at the Bokbunja substation of the Gochang Agricultural Center (Table 1). Total genomic DNA was extracted from pulverized leaf samples using Plant DNAzol Reagent (Invitrogen, Carlsbad, CA, USA) according to the supplier’s protocol. The DNA concentration was determined using an ultraviolet-visible spectrophotometer (ND-1000; NanoDrop, Wilmington, DE, USA). The final concentration of each DNA sample was adjusted to 20 ng/µL in TE buffer before implementing the PCR procedure.

### 3.2. Construction of an SSR-Enriched Library and Primer Design

A modified biotin-streptavidin capture method was used to construct an SSR-enriched library of *R. coreanus* [27]. Genomic DNA of *R. coreanus* was digested with restriction enzymes (*Alu*I, *Dra*I, *Hae*III, *Rsa*I, *Eco*RV, and *Nru*I), and the fragmented DNA was eluted on a 1.5% agarose gel; the fragments ranged in size from 300 to 1500 bp. After adaptor ligation and pre-amplification, the DNA mixture was hybridized with the following biotin-labeled oligonucleotides: (GA)_20_, (CA)_20_, (AT)_20_, (GC)_20_, (AGC)_15_, (GGC)_15_, (AAG)_15_, (AAC)_15_, and (AGG)_15_. The hybridized DNA fragments were recovered with streptavidin-coated magnetic beads (Promega, Madison, WI, USA). The fragments were cloned by TOPO TA cloning (Invitrogen). Recombinant colonies, identified as white colonies on an LB plate containing ampicillin, were sequenced using an ABI3100 DNA sequencer (Applied Biosystems, Foster City, CA, USA). SSR Manager [28] was used to identify the SSR motif and to design primers flanking these regions.

### 3.3. PCR Amplification and Genotyping

The M13-tailed primer method, in which the M13 sequence is attached to the 5'-end of the forward primer, was used to determine the sizes of the amplified products, as described by Schuelke [29]. PCR amplification was performed in a total volume of 20 µL, which consisted of 50 ng of genomic DNA, 2 pmol of a specific primer, 4 pmol of the fluorescently labeled M13 universal primer (5'-TGT AAA ACG GCC AGT-3'), 6 pmol of a normal reverse primer, 2.0 µL of 10× PCR buffer (Solgent, Daejeon, Korea), 1.6 µL of a dNTP mixture (2.5 mM), and 1 U of Taq polymerase (Solgent). The reaction conditions were: 94 °C for 3 min; 30 cycles of 94 °C (30 s), 56 °C (45 s), and 72 °C (1 min); 10 cycles of 94 °C (30 s), 53 °C (45 s), and 72 °C (1 min); and a final extension step at 72 °C for 10 min. PCR was performed in a PTC-200 thermocycler (MJ Research, Waltham, MA, USA), and the amplified fluorescently labeled PCR products were resolved on an ABI PRISM 3130xl Genetic Analyzer (Applied Biosystems) with an internal size standard (Genescan-500 ROX). Fragments were sized and scored as alleles using GeneMapper ver. 4.0 (Applied Biosystems).

### 3.4. Analysis of Transferability, Genetic Variability, and Evolutionary Relationships

The 97 microsatellite loci with a reproducible amplicon in *R. coreanus* were cross-amplified in other *Rubus* species, and their transferability was analyzed. PCR and resolving the amplified products were performed as described above. Genotypes of the 48 *Rubus* accessions were based on the 29 polymorphic microsatellite markers, and these DNA profiles were used to analyze genetic variability and construct a phylogenetic tree. PowerMarker (ver. 3.25) [30] was used to measure the genetic variability at SSR loci among *Rubus* species. A genetic distance matrix was created as a function of shared alleles [31]. The construction of a phylogenetic tree and bootstrapping were performed using PowerMarker (ver. 3.25) according to the un-weighted pair-group method with arithmetic averages. A consensus tree was created using the PHYLIP software package (ver. 3.695) [32].

## 4. Conclusions

We created 29 new polymorphic microsatellite markers based on the 97 transferable microsatellite markers in the *Rubus* species. The genetic diversity index and clustering results show that these markers are informative for genetic assessments of *R. coreanus* and other *Rubus* species. These new microsatellite markers can be used for genetic map construction, molecular breeding, and germplasm management of *Rubus* species.

## Supplementary Materials

Supplementary materials can be accessed at: http://www.mdpi.com/1420-3049/20/04/6432/s1.
